# Single unit activities recorded in the thalamus and the overlying parietal cortex of subjects affected by disorders of consciousness

**DOI:** 10.1371/journal.pone.0205967

**Published:** 2018-11-07

**Authors:** Lorenzo Magrassi, Antonio G. Zippo, Alberto Azzalin, Stefano Bastianello, Roberto Imberti, Gabriele E. M. Biella

**Affiliations:** 1 Neurochirurgia, Dipartimento di Scienze Clinico-Chirurgiche, Diagnostiche e Pediatriche, University of Pavia—Fondazione IRCCS Policlinico S. Matteo, Pavia, Italy; 2 Istituto di Genetica Molecolare IGM-CNR, Pavia, Italy; 3 Istituto di Bioimmagini e Fisiologia Molecolare, CNR, LITA Bldg, Segrate, Italy; 4 State University of Pavia, Dept. of Brain and Behavioral Sciences, Neuroradiology Department—C. Mondino National Neurological Institute, Pavia, Italy; 5 Phase I Clinical Trial Unit and Experimental Therapy, Fondazione IRCCS Policlinico S. Matteo, Pavia, Italy; University of Regensburg, GERMANY

## Abstract

The lack of direct neurophysiological recordings from the thalamus and the cortex hampers our understanding of vegetative state/unresponsive wakefulness syndrome and minimally conscious state in humans. We obtained microelectrode recordings from the thalami and the homolateral parietal cortex of two vegetative state/unresponsive wakefulness syndrome and one minimally conscious state patients during surgery for implantation of electrodes in both thalami for chronic deep brain stimulation. We found that activity of the thalamo-cortical networks differed among the two conditions. There were half the number of active neurons in the thalami of patients in vegetative state/unresponsive wakefulness syndrome than in minimally conscious state. Coupling of thalamic neuron discharge with EEG phases also differed in the two conditions and thalamo-cortical cross-frequency coupling was limited to the minimally conscious state patient. When consciousness is physiologically or pharmacologically reversibly suspended there is a significant increase in bursting activity of the thalamic neurons. By contrast, in the thalami of our patients in both conditions fewer than 17% of the recorded neurons showed bursting activity. This indicates that these conditions differ from physiological suspension of consciousness and that increased thalamic inhibition is not prominent. Our findings, albeit obtained in a limited number of patients, unveil the neurophysiology of these conditions at single unit resolution and might be relevant for inspiring novel therapeutic options.

## Introduction

Subjects in vegetative state/unresponsive wakefulness syndrome (VS/UWS) [1] and in minimally conscious state (MCS) [2] are characterized by preserved arousal without (VS/UWS) or inconsistent (MCS) behavioral evidence of awareness of self and surrounding. Unconsciousness in these patients is correlated with decreased positive connectivity within the default mode network and abnormally increased positive connectivity between this and the task-positive network [3], while the activities of these two networks are anticorrelated in normal subjects [4].

These changes in functional connectivity are not exclusively generated by cortical damage but also reflect changes in the activity of the thalamus and basal ganglia [5,6]. Widespread structural damage of the brain is highly variable in patients affected by disorders of consciousness (DOC), although damage to the thalamus and/or its connections is invariably present [7]. Thalamic damage followed by atrophy is usually more extensive in trauma patients [8].

The important role played by the thalamus and its reciprocal connections with the frontal and parietal lobes and the basal ganglia in maintaining consciousness is also suggested by studies showing increased neural connectivity between the intralaminar thalamus and the anterior frontal cortex during recovery of consciousness [9]. The evidences supporting the important role of the alterations of thalamus and its reciprocal connections with the cortex in DOC patients come mainly from post-mortem or non invasive studies based on functional magnetic resonance (fMRI).

The application of advanced magnetic resonance techniques to the study of DOC has been a major advance and due to its non invasive nature allowed the introduction of control groups and larger populations of subjects however fMRI techniques are intrinsically limited in spatial and temporal resolution and often requires anesthesia of the patients affected by DOC to reduce head movements [10].

Electroencephalography possess greater temporal resolution than MRI but only extracellular single unit recordings with microelectrodes allows both maximal temporal and spatial resolution [11]. However, microelectrode recordings are invasive and difficult to perform in humans unless they are part of the neurophysiological studies necessary for selected neurosurgical procedures [12,13].

Due to the lack of single unit recordings from the cortico-thalamic structures in subjects affected by DOC it is still unknown whether unconsciousness in these subjects is related to the same changes in neural activity that were described during the physiological transition from consciousness to unconsciousness.

In humans deactivation of the medial thalamus (MT) precedes cortical deactivation into non-rapid eye movement sleep (NREMS) [14]; however, intracellular recording studies *in vivo* in the mouse indicate that wakeful synaptic dynamics in the cortex can occur independently of thalamic afferents [15].

One hypothesis is that DOC depend on an excessive inhibitory input from the globus pallidus internus (GPi) to the MT which reduces its excitatory output to the cortex [16]. However, increased inhibition of the MT by GPi is present in Parkinson disease and obsessive compulsive disorders which are not associated with DOC [17,18].

To help clarify the role of the MT in DOC we recorded single unit activity from the centromedian-parafascicular (CM-Pf), central-lateral (CL) and paralaminar part of mediodorsal (plMD) nuclei of the thalamus of patients in VS/UWS or MCS under light anesthesia during placement of bilateral thalamic electrodes for deep brain stimulation.

## Materials and methods

### Subjects

EEG and microelectrode recordings were obtained during neurosurgical operations performed on all patients participating to the study CATS (ClinicalTrials.gov identifier NCT01027572). We already published complete clinical information on patient status, complications and outcomes of all patients participating to the study CATS [19] and we are now presenting the results of the studies we conducted on the neurophysiological recordings obtained in those patients. Research protocols were approved by the institutional review board of the Fondazione IRCCS Policlinico S. Matteo (Pavia, Italy) and later revised and approved by the institutional review boards of the Fondazione IRCCS Maugeri (Pavia, Italy) and Fondazione IRCCS Istituto Neurologico Nazionale C. Mondino (Pavia, Italy) which joined the study. Informed consent was obtained for all patients from the patients’ legal representatives and evaluation of the patients for enrolment into the study started upon request by the person with legal responsibility for the patient. The study was conducted in accordance with the Declaration of Helsinki. We adopted the Italian version of the Coma Recovery Scale-Revised (CRS-R) for assessing the severity of the DOC [20]. Three male patients two in VS/UWS indicated as VS1 and VS2 and one in MCS indicated as MCS, were the subjects of bilateral thalamic microelectrode recordings during surgery for the implantation of bilateral stimulating electrodes in the thalamus for deep brain stimulation.

VS1 lost consciousness after a closed head injury during a car accident and never regained it. He remained eight years in VS/UWS before entering the study when he was 29-year-old. During this period he developed generalized spasticity with bilateral myoclonic mass movements that could be evoked or exaggerated by minor tactile stimulation or environmental sounds (startle myoclonus). His CRS-R score before electrode implantation was 6.

VS2 had bilateral frontal acute subdural hematomas associated with multiple cortical lacerations as a result of a car accident. He underwent surgical evacuation of the hematomas and decompressive craniectomy, within four months after the initial surgery, he underwent cranioplasty with autologous bone. He was 23 year old when, two years and 10 months after the initial car accident, he underwent bilateral implantation of stimulating electrodes into the thalami. His CRS-R score at the time of surgery was 8 and he had generalized spasticity and evoked myoclonus.

MCS as a consequence of a car accident had head injury with multiple lacerations in frontal lobes bilaterally and the right parietal lobe. He was treated conservatively and after an initial improvement from VS/UWS to MCS (he sometimes moved his right hand on request) he remained stable for the rest of the two years and four months elapsed before surgery. At the time of surgery he was 58-year-old and his CRS-R score was 14.

Pre-operative MRI scans in all patients of the present study showed that the medial thalamus was not damaged.

### Anesthesia

General anesthesia was explicitly requested by the review board. Induction of anesthesia and intubation were performed with propofol, fentanyl (50–100 mcg) and vecuronium. The patient was mechanically ventilated and anesthesia was maintained with sevoflurane and vecuronium, implemented with fentanyl at the time of drilling and during the tunnelization of the electrodes. When microelectrode recordings were performed during surgery, the end-tidal concentration of sevoflurane was reduced to 0.5–0.7%, a concentration well below the MAC_90_ for this anesthetic vapor. Monitoring included continuous electrocardiography with automatic ST segment analysis, invasive arterial blood pressure measurements, pulse-oximetry, capnometry and recordings of esophageal temperature and urine output.

### Surgical approach and electrode targeting

After positioning the CRW (Integra—Radionics, Burlington, MA 01803 USA) stereotactic headframe under local anesthesia the patient underwent intravenous contrast MRI and CT scans. After fusion of the MRI with the CT scan the positions of the anterior and posterior commisures were determined on a mid-sagittal MRI section and the images registered to an electronic version of the Schaltenbrand and Wahren stereotactic atlas (Integra—Radionics, Burlington, MA, USA). Due to brain atrophy and alterations peculiar to each single patients resulting from traumatic brain injury the atlas data could not be used directly for choosing the trajectories of the microelectrodes and we targeted the base of the central-medial thalamus corresponding to the paralaminar part of the mediodorsal nucleus, the CL nucleus and the CM-pf nucleus as previously indicated by Schiff et al. [21]. We started from an entry point located in the homolateral parietal cortex through a burr hole with a diameter of 5 mm. Five microelectrodes (microTargeting model 22670, FHC Bowdoinham, ME, USA) distant 2 mm axis to axis in 1 patient and 4 microelectrodes in 2 patients were guided to the target using the "Ben Gun" tool with five parallel channels. Electrodes were advanced in 1 mm steps from 15 mm before the target to 10mm beyond it. After recording the microelectrodes were removed and through the trace of 1 microelectrode 1 quadripolar electrode (model 3387, Medtronic Italia S.p.A. Sesto San Giovanni, Italy) was positioned with the second contact from the tip corresponding to the target position.

### Neuroradiological investigations

In all patients we obtained a postoperative CT scan in the 12 hours following the initial surgery to check for possible complications. CT scans were also fused to the MRI images of the patients to check for electrode positions. Control MRI were also obtained in the following weeks with a 1.5 tesla apparatus. For each patient T1 (with and without contrast) and T2 weighted images in axial, coronal and sagittal orientations were acquired and used for all the neuro-anatomical evaluations.

### Microelectrode recordings and spike sorting

Microelectrode recordings were performed using the Isis Mer system (Inomed Medizintechnik GmbH, 79312 Emmendingen, Germany) at 25 KHz of sampling rate. We sorted each unit by using an opportunely tuned version of the "wave_clus" framework [22]. Spikes were detected by means of a threshold set to the medians of the filtered signals divided by 0.6745. Waveforms were extracted and featured by scores obtained with principal component analysis, and components were selected through a Gaussian fit test, and over-clusterized by the k-means algorithm and partially merged post-hoc. Best parameters for clustering were obtained through local searching by running the algorithm 100 times and randomly varying one parameter at each run as previously described [23]. After each run the solution was evaluated against the previous run. If an improvement was observed the modification was maintained; otherwise a new change was performed on the previous parameter set [23].

To detect burst events, we used a threshold crossing approach [24] set to identify even the smallest trains of action potentials as a burst. We applied a filter Z on the extracellular recording x, according to the following steps:

1. *Y* = (Δ*x*)^2^
2. *K* = *G*(*μ* = 0,*σ* = 3)
3. *Z* = *conv*(*Y*,*K*)

where Δ represents the centered discrete first derivate, G is the Gaussian Kernel with the mean equal to μ and standard deviation set at σ, “conv” is the convolution function. The threshold was empirically chosen as the median of the distribution of the instantaneous means of the raw signal divided by 0.44 (S1 Fig).

### Cross-correlation analysis

In order to assess the extent of correlation between the firings of thalamic and cortical recorded units, we used the unbiased normalized “xcorr” MATLAB function by windowing signals into 100 ms bins. The absolute value of the maximum of estimated cross-correlation was computed for each bin and the corresponding lag obtained. We estimated the threshold for an acceptable cross-correlation between thalamic and cortical unit activities by evaluating the extent of random correlation in the recording tracks obtained simultaneously from the parietal cortex and the thalamus, by shuffling the binarized spiking activity time series (1 for a spike, 0 otherwise) and subsequently computing the maximum cross-correlation emerged between all thalamic and cortical neurons [25]. Eventually, we averaged such estimation over 10,000 repetition of the previous step in order to unbias the accuracy. We obtained that in 499 cases (out of 10,000, i.e. the 5% at most), the maximal cross-correlation was above 0.05 and below 0.1466, which was rounded to 0.15 and used as the threshold level for cross-correlation significance, in all other cases the value obtained was below 0.05.

### EEG analyses

EEG recordings from 4 leads (2 frontal and 2 occipital) were also obtained using a multichannel digital electroencephalograph (System Plus; Micromed, Italy) with a maximal sampling rate of 2048 Hz and an analog to digital converter resolution of 16 bits. EEG signals were analyzed by EEGLAB: an open source toolbox for analysis of single-trial EEG dynamics [26].

EEG signals and thalamic large field potentials obtained from the electrodes implanted in the thalami were first filtered in the frequency range (1–40 Hz) in which we found ~99% of the signal power spectrum and then further parted by a finite impulse response filter (0.3 Hz of width) into five bands: delta (1–4 Hz), theta (4–8 Hz), alpha (8–13 Hz), beta (14–30 Hz) and low gamma (30–40) Hz. The fast Fourier transform of the EEG and thalamic local field potentials (LFP) power spectra were estimated by the spectopo.m function of EEGLAB. To study the coupling between EEG and the occurrence of spikes in the microelectrodes, we followed a previously validated approach [27] that allows calculation, for each unit, of the distribution of the EEG phases corresponding to each spike occurrence. We computed the EEG phase corresponding to each spike by extracting for each EEG band the instantaneous phase by the argument (“angle” function in Matlab) of the Hilbert transform (“Hilbert” function in Matlab) and we discretized the range of the possible values of phase [*0*, *2*π] into 4 classes (A: [0, π*/2*], B: [π*/2*, π], C: [π, *3*π*/2*], D: [3π*/2*, 2π]). We used the Rayleigh test of nonuniform angular distribution [28] to statistically test the hypothesis that spikes were not uniformly distributed in the EEG phases.

### Cross-frequency coupling

Phase-amplitude CFC, which refers to the statistical dependence between the phase of a low-frequency brain rhythms and the amplitude of the high-frequency brain activity component, is the most important CFC feature [29]. Starting from thalamic LFP and the grand average of all EEG electrode recordings, we calculated the phase-locking value (PLV) where the high-frequency amplitudes are filtered to extract the low-frequency phase which is then compared with the low-frequency phase extracted from the raw signal. Formally if ϕ_δ_(*n*) and ϕ_α_(*n*) are, respectively, the phases of delta and alpha oscillations in window *n* then the PLV is defined as:

$$PLV=\left|\frac{1}{N}\sum_{n=1}^{N}e^{i(\phi_{\delta }(n)-\phi_{\alpha }(n))}\right|$$

where *e* is the Napier constant, *N* is the number of signal samples within the window and *i* is the imaginary unit [30,31]. Phase was computed as the argument of the Hilbert transform and a bandpass FIR filter was used to extract components of interest. We compared the delta (1–4 Hz) and theta (4–8 Hz) bands with alpha (8–12 Hz), beta (12–25 Hz) and gamma (25–40 Hz) bands. Values were computed by windowing signals in 500 ms bins.

### Statistical analysis

The following statistical tests were implemented where indicated in the text. The results were calculated using MedCalc for Windows, version 14.8.1 (MedCalc Software). Pearson's chi-squared test was used as test of homogeneity and independence, and Wilcoxon non-parametric rank sum test was used in non-categorical comparisons. Two-way anova was used to assess whether burst durations changed at different recording depth from the cortical surface. In all hypothesis tests, the level of significance was 0.05 except when downgraded by Bonferroni correction. The Rayleigh test was calculated using the circular statistic toolbox of Matlab. Analyses of phase-amplitude coupling were statistically supported by an ad-hoc test where each of the five frequency bands (delta, theta, alpha, beta, gamma) populated two diverse sets, one for the phase and one for the amplitude for each patient. E.g. MCS1 phases were collected into a 5-column table (one for each band) and the significant differences among bands extracted by Kruskal-Wallis test. If the returned p-value was smaller than 0.05, the post-hoc Dunn test selected the bands that effectively showed a significant difference. Similarly, MCS1 amplitudes underwent the same procedure. Eventually, when both tests returned significant bands for the same patient we considered significant the phase-amplitude coupling.

## Results

### Single units recorded in the thalamus of VS/UWS and MCS patients

**Fig 1:**
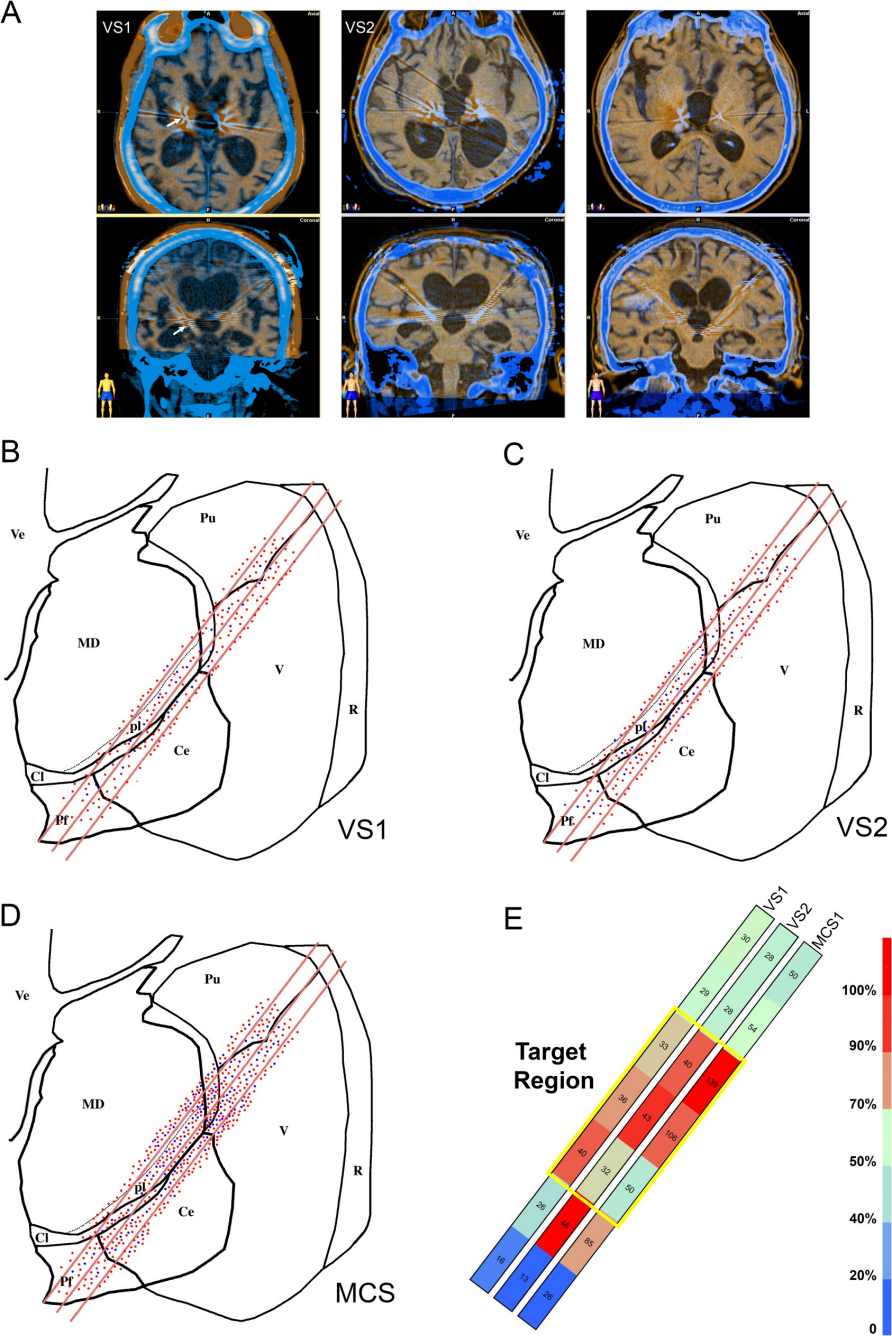
Microelectrode recordings from the thalamus of patients affected by DOC. (a) Early postoperative TC fused to MRI T1 weighted images showing the position of the definitive electrodes for thalamic stimulation in all patients (E: electrode). The stimulating electrodes were implanted following the trajectory of one of the recording microelectrodes thus their position marks the intra-thalamic region of the microelectrode recordings. In the upper row we show the axial images corresponding to the patients, in the lower row the coronal ones. The withe arrows in VS1 indicate the position of the electrode in the thalamus. (b-d) Topographic distribution of the 950 thalamic units recorded in the 2 VS/UWS patients (b: VS1, c: VS2) and in the MCS patient (d: MCS) shown on coronal view of the left thalamus at the level of the microelectrode trajectories. Only the last 20 mm of the trajectories of the 3 microelectrodes contained in the plane of the section are outlined (red segments). All recorded units are indicated at their position. Units recorded by the electrodes contained in planes parallel to that outlined in the figure were projected to the represented plane and all units recorded in the right thalamus were also projected to the corresponding position of the left one. Red dots: SN, blue dots: BN. Pf: parafascicular nucleus, MD: mediodorsal nucleus, pl: Paralaminar part of mediodorsal nucleus, Cl: central lateral nucleus, Ce centromedian nucleus, V: ventral nuclear group, Pu: Pulvinar, R: reticular nucleus. (e) Heatmap of the distribution of the recorded units in the three patients. The represented recordings were obtained every 1mm in 21 steps starting from 0 located dorsally and lateral in the thalamus to 20, ventral and more medial close to the third ventricle. Recordings were grouped in 7 bins containing the cells collected by multiple electrodes bilaterally in 3 recordings step each. The color of each bin represent the percentage of total neurons recorded present in each bin, the numbers of neurons are written inside each bin. The yellow rectangle outlines the 2D projection of the 3D region where, anatomically, the microelectrodes had the maximal chance of recording from the nuclei representing the intended the target for stimulation. A color scale is provided with the indication of percentages corresponding to each color.

Analyzing all microelectrode recordings obtained during the prospective multicenter study CATS [19], we identified 440 spontaneously active units in the thalami of the VS/ UWS patients (210 in VS1 and 230 in VS2) and 510 units in the MCS patient. This difference suggests a significantly higher level of ongoing activity in MCS (χ^2^ = 177.684; DF:2; P < 0.001). The appropriateness of the stereotactically guided trajectories of the microelectrodes was also confirmed by imaging the positions of the stimulating electrodes within 12 hours from surgery (Fig 1A). The spatial distribution of the active units was similar in all patients (Fig 1B–1D) suggesting that the higher number of units recorded in MCS compared to VS truly reflected a decreased density of spontaneously active units in VS and not an artifact related to some undetected change in the trajectory of the microelectrodes. In all patients the number of active units was higher close to the expected location of the thalamic nuclei that were selected for stimulation than at the extremes of the 20 mm intra-thalamic trajectories (Fig 1E).

### Spiking vs. bursting neurons

**Fig 2:**
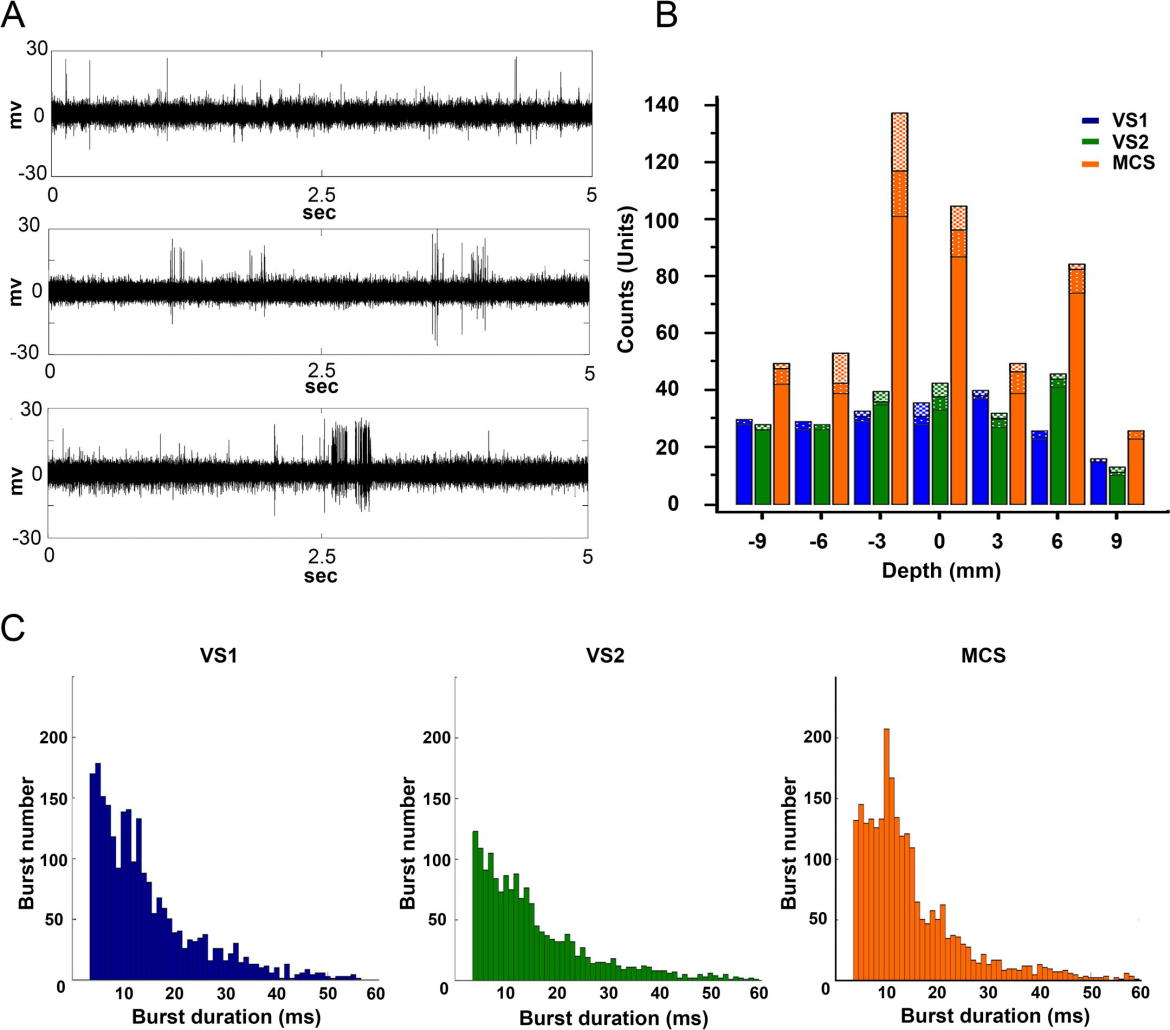
Characterization of the single unit activities recorded in the thalami. (a) Examples of activities recorded in the thalamus of the patients affected by DOC before sorting. The duration of all traces is 5 sec. The upper trace from VS1, shows mainly SN discharging randomly. The middle trace from VS2, shows randomly BN, with burst duration less than 15 ms. The lower trace from MCS, shows randomly BN, with two bursts lasting longer than 150 ms (MCS). (b) Histograms showing the distribution of burst durations in each patient. Two-way anova test showed that bursts durations were different at each depth (F = 2.85, P = 0.0165). Burst durations were significantly different when we compared MCS and VS1 (Wilcoxon ranksum test with Bonferroni correction: ranksum = 68; P = 0.0018) and MCS and VS2 (Wilcoxon ranksum test with Bonferroni correction: ranksum = 85, P< 0.0001), this was true independently from the depth of the recording. (c) Histogram showing the distribution of the single units recorded at different depths in the thalami of the 3 patients VS1 (blue), VS2 (green) and MCS (red). The distribution of the recorded units was significantly different in MCS, VS1 and VS2 (χ^2^ = 38.148; DF:12; P < 0.001). Units were classified according to their prevalent activity into SN and BN, and BN units were further subdivided into units with burst lasting for <14 ms (squared texture) or >13 ms (stippled texture). Differences in the distribution of the 3 class of units according to the level explored was significant only in MCS (χ^2^ = 25.395; DF:12; P < 0.013) while in VS1 and VS2 units were uniformly distributed at all levels.

Previous microelectrode recordings from the MT of awake patients [32,33] and recordings in animals [34] indicated that MT neurons can be classified into two classes: spiking neurons (SN) firing single action potentials and bursting neurons (BN) firing trains of action potentials of different durations (Fig 2A). There were significantly more SN and BN in the thalami of the MCS patient than in VS patients (χ^2^ = 10.852; DF:2; P < 0.0044) (Fig 2B). Moreover, the distribution of burst duration was different in VS vs. MCS patients (Fig 2C).

Extracellular recordings are unable to document the level of inhibition of the thalamic neurons directly. However, bursting activity in the thalamus depends on the de-inactivation of low-threshold T-type Ca^2+^ channels by membrane hyperpolarization [35], so the number of BN is an indirect measure of the inhibition of the neurons in the area. This assumption is supported by microelectrode recordings in the MT of Parkinsonian patients showing a significant increase in the number of BN due to increased inhibitory input from the GPi [18,33]. Increased bursting activity in primary and secondary nuclei of the thalamus due to increased inhibitory inputs from different sources is also typical of NREMS [34,36]. BN can be present independently of the level of consciousness in the CL [33,37], and less frequently in the CM-Pf nuclear complex where they are modulated by selective attention [32]. In our recordings, fewer than 17% of the neurons had consistent bursting activity while 83% were SN. It is unlikely that the low number of BN was due to the light sevoflurane anesthesia. In the CM nucleus of mice the number of spikes in SN and the number of spikes per bursts in BN was reduced only by higher concentrations of sevoflurane higher than those we used in the present study [38]. Moreover, sevoflurane would have induced a relative increase in the number of BN over SN and not the opposite, as we found.

### Correlation between neural activities in the thalamus and the cortex

**Fig 3:**
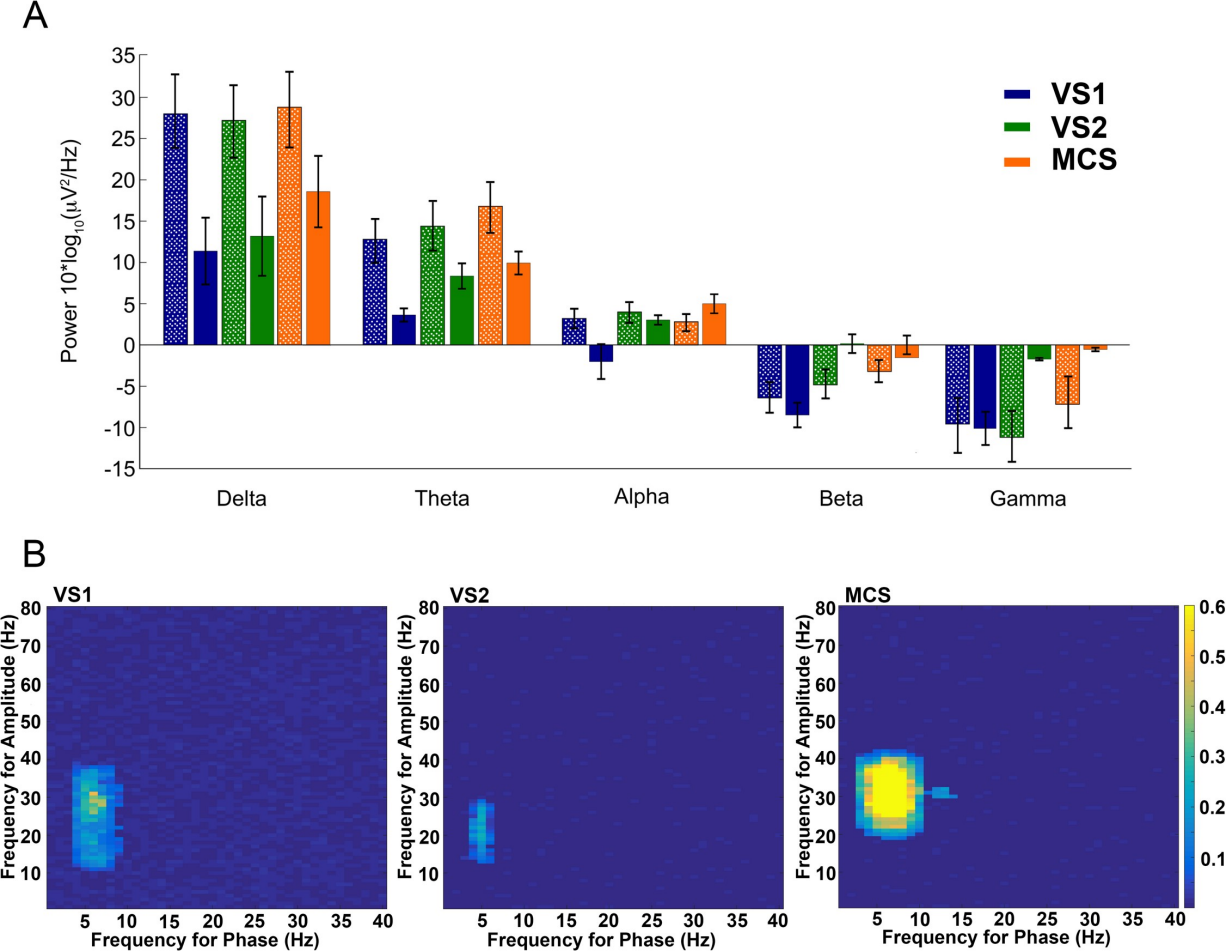
Characterization of thalamo-cortical coupling. (a) Bar graph displaying the power spectral analysis of the LFP (stippled texture) recorded from the thalami of VS1 (blue), VS2 (green) and MCS (red) and the corresponding power spectrum obtained from the EEG (no texture) simultaneously recorded in the same patients. All recordings were obtained with the patient resting quietly under normal ambient lighting without active auditory or tactile stimulations exept for background pressure from the bed and bedclothes. As expected delta and theta activity prevails both in the thalamus and the cortex, with the larger power in the thalamic spectra mainly resulting from the attenuation of the cortical activity in the EEG due to the extracranial position of the recording electrodes. Columns correspond to the average power from multiple recordings, error bars: standard error. (b) Comodulograms showing modulation strength as a function of frequency for amplitude and frequency for phase between thalami and cortex of VS1, VS2 and MCS. We obtained all the analyzed recordings with the patients in an apparent resting condition without environmental or background sensory stimulation. Only in MCS was there significant modulation (phase: H = 21.342, P < 0.0001; amplitude: H = 17.920, P < 0.001; Kruskal-Wallis and Dunn tests as post-hoc) of the cortical low gamma activity power by thalamic theta-alpha coupling indicating CFC between thalamus and cortex.

In order to study the influence of the MT on the cortex in DOC patients we compared the power spectra obtained from the EEG or from the large field potentials recorded in the thalamus (Fig 3A) and tested for thalamo-cortical (TC) phase-amplitude co-modulation.

Cross-frequency coupling (CFC) among different brain areas is present during neuronal computation, communication and learning and seems important to orchestrate brain networks at different spatiotemporal scales [29]. CFC between TC and cortico-thalamic activities is present in humans as demonstrated by direct recording in epileptic patients [39] and by magnetoencephalography [40]. We found strong theta/alpha to low gamma CFC in MCS whereas this was absent in VS2 and minimal in VS1 (Fig 3B).

**Fig 4:**
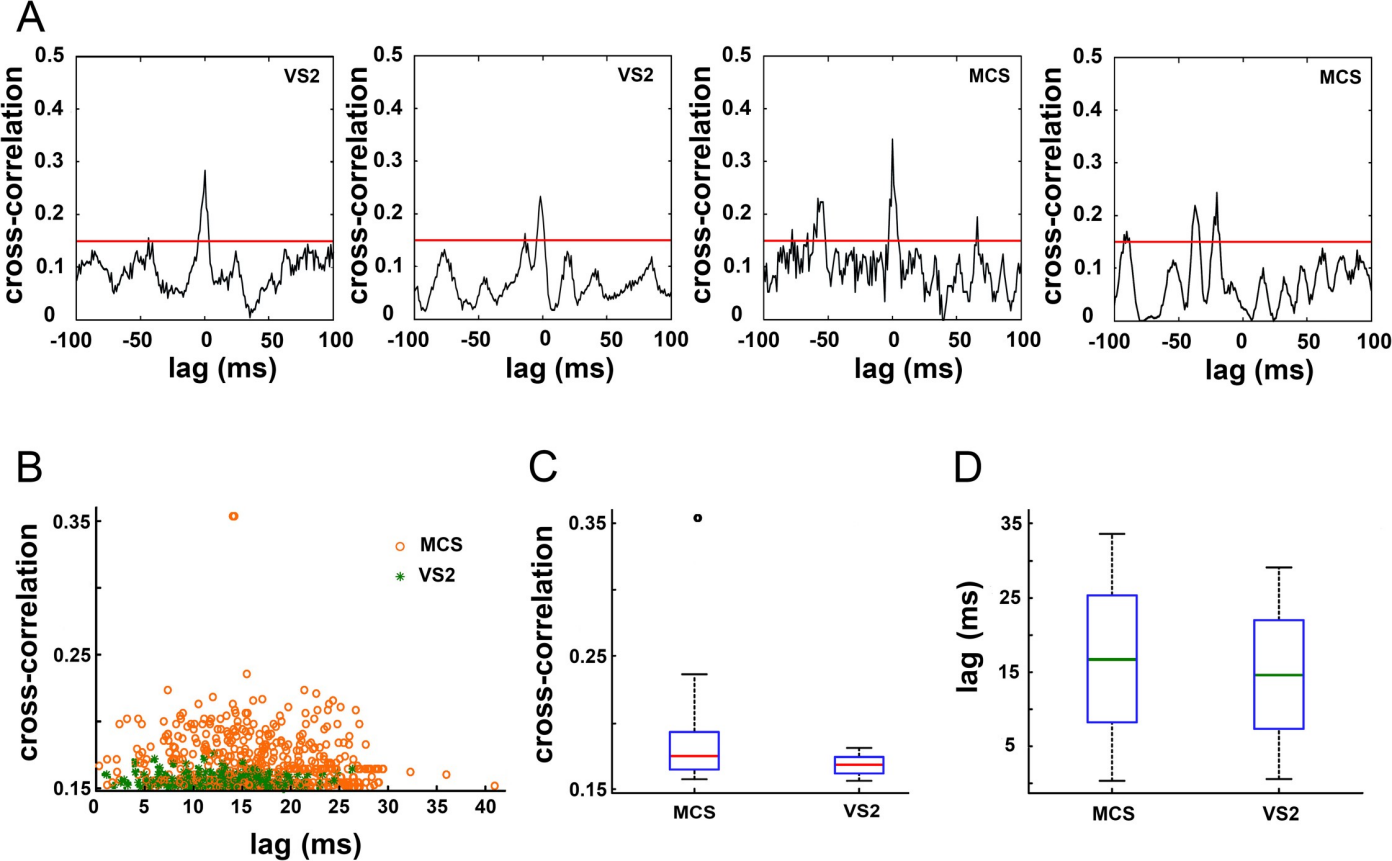
Characterization of thalamo-cortical coupling. (a) Four examples of typical cross correlograms thalamo-cortical pairs recorded in VS2 (first and second cross-correlograms) and MCS1 (third and fourth cross-correlograms). The short lag (< 3 msec) of the peek correlation shown in the first three correlograms suggests the persistence of functional monosynaptic cortico-thalamic connections. The red line at 0.15 indicates the threshold level for cross-correlation significance. This value was obtained after extensive resampling and shuffling of the recordings as described in the methods. (b) Diagram showing the maximal cross-correlations between the activities of single units in the thalamus and the homolateral parietal cortex and the absolute lag corresponding to the cross correlation. Background correlation threshold was 0.15. Each circle represent the cross correlation between one thalamic unit and the cortical unit maximizing the cross-correlation. Orange circles: MCS and green asterisks: VS2. (c) Distribution of the cross-correlation amplitudes: red line, median; blue box, interquartile range; whiskers, range from 1–99 percentiles. (d) Distribution of the absolute lags: green line, median. No significant difference is visible between MCS and VS2.

We also obtained synchronous microelectrode recordings from the parietal cortex (PC) near the entry point of the microelectrodes, in VS2 and MCS. Although TC coupling varies in different cortical areas [33,41], TC activity has widespread influences on PC even outside the primary sensory cortex [41,42]. We cross-correlated single unit activities in the PC and in the MT, and we found that 27 (8 in VS2 and 19 in MCS1) of 137 PC neurons had cross-correlation values above chance level (0.15) (Fig 4A and 4B). We found both in VS2 AND MCS1 several units reaching the peak of cross-correlations at very short lags (< 3msec) suggesting the persistence of monosynaptic connections between the thalamus and the parietal cortex (Fig 4A and 4B).

### Differences between VS/UWS and MCS in the phase of EEG synchronous neural discharge

**Fig 5:**
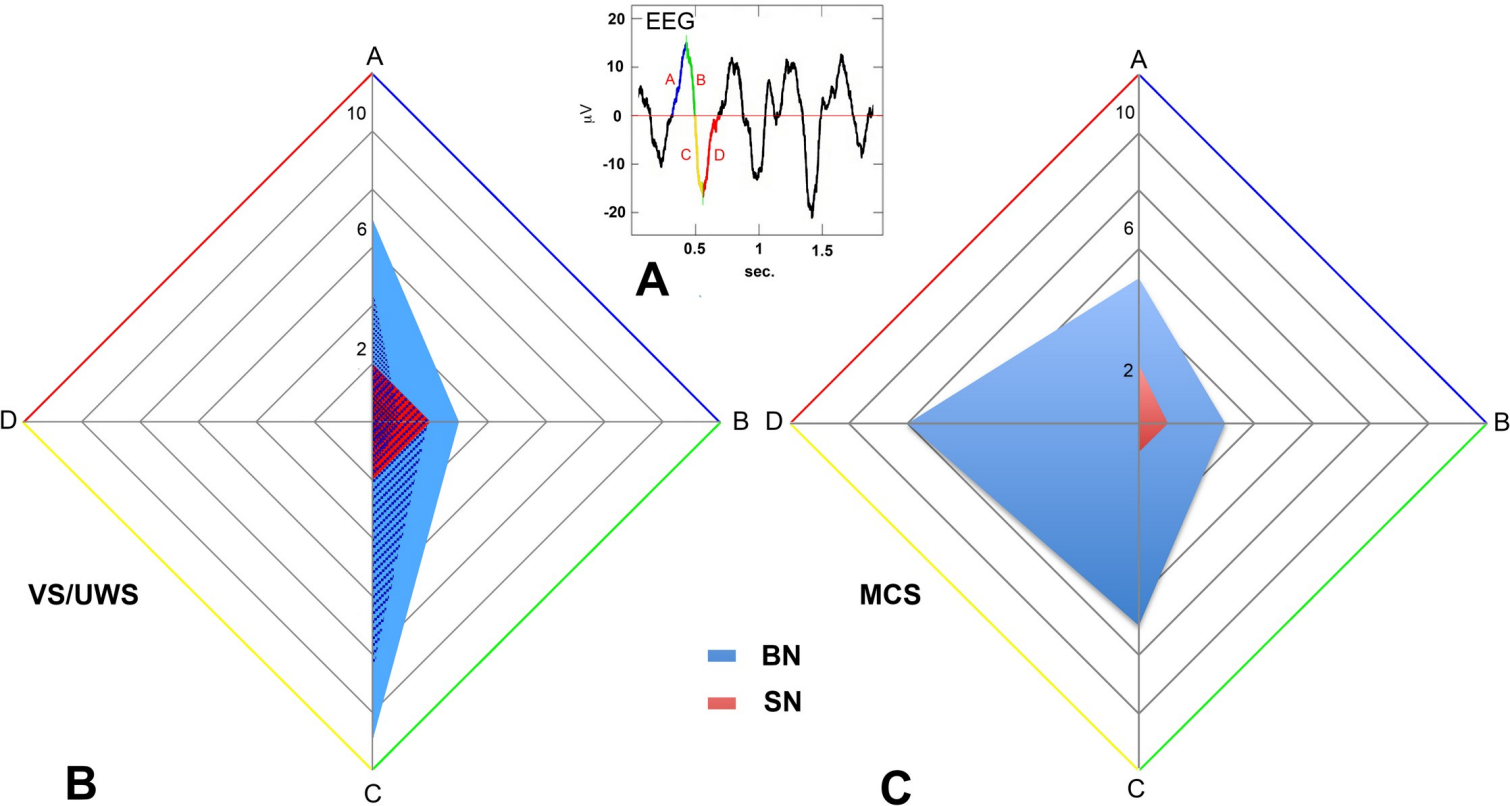
(a-c) Single thalamic units discharging in phase with the EEG. (a) Superimposed on a wave of the EEG trace are indicated the 4 phase bins: A (blue), B (green), C (yellow), and D (red), used to classify thalamic units firing in phase with the EEG. A and B correspond to the up deflection while C and D correspond to the down deflection of the EEG wave. (b-c) Radar plots of the number of units firing synchronously with the four phases of the EEG wave shown in a. In b are represented both the total number of the spiking (red) and bursting (blue) units recorded in VS/UWS patients and the contributions of the single patients: circles represent the units firing synchronously with the EEG recorded in VS1, while the squares indicate the units recorded in VS2. Overall, BN (BN firing in phase n. 44, 27.3%, BN total n. 161) were more likely (χ^2^ = 169.229; DF:1; P < 0.0001) firing in phase with the EEG than SN (SN firing in phase n. 10, 1.3%, SN total n. 789). This was true in both VS/UWS and MCS patients. In VS/UWS patients (b) we found no unit firing synchronously with phase D of the EEG while in the MCS patient (c) we detected 8 BN in D. The difference in the distribution of units firing synchronously to the different EEG phases among VS/UWS and MCS patients is statistically significant (χ^2^ = 14.452; DF:6; P < 0.025).

Altogether, the above results indicate that cortico-thalamic coupling is still present in MCS and reduced but not completely lost in VS/UWS. Persistent cortico-thalamic coupling was also demonstrated by the presence of units discharging in time with the cortical EEG [37,43]. We found 27, 16 and 11 units whose firing was coupled to the EEG in the thalami of MCS, VS1 and VS2, respectively. In all patients these cells were more frequent at or around the target than at the extremes (χ^2^ = 20.167; DF:1; P < 0.0001). The proportions of cells firing in time with the EEG and those that did not, were similar in all patients (χ^2^ = 1.960; DF = 2; P = 0.375). During natural sleep states cortical and thalamic neurons that discharge in phase with the EEG tend to fire in coincidence with the negative component of the depth EEG [43,44] that in humans correspond to the positive component of the surface EEG [42]. In our study, we found in all patients neurons discharging in phase with both the surface EEG positive and negative components (S1 Table). However, in VS/UWS patients SN discharge was limited to the initial phase of the EEG negative deflection while in MCS active neurons were found through the entire negative deflection (χ^2^ = 9.552; DF: 3; P = 0.0228) (Fig 5A and 5B**)**.

## Discussion

Several important conclusions can be drawn from our results. First, the spontaneous single unit activity present in the MT of patients with DOC is dominated by SN and not BN. This is the opposite of what happens when consciousness is physiologically reversibly suspended in humans [36] and animals [34]. This indicates that these conditions differ from physiological suspension of consciousness.

Second, the activity of MT cells we recorded in DOC seems more compatible with widespread damage and decreased excitatory inputs than exaggerated inhibition. This is suggested by the low number of BN we found in DOC patients, while BN are increased in Parkinson disease in which increased inhibitory input to the thalamus has been documented [18,33]. Indications of decreased excitatory input and widespread damage also come from the observation that the number of active units was lower in VS/UWS patients compared to MCS. An inverse relationship between severity of the pathology and the number of active units in the thalamus has also been described in patients that were not affected by DOC, and is considered an indication of damage to the thalamus and its connections [45]. Furthermore, the limitation of significant TC CFC to MCS and the presence of only a few MT neurons whose activity correlated with cortical neuron activity or was synchronized with the EEG is another indication of damage more than excessive inhibition. Interestingly, moderate cortical cooling of the cortex that induces a reversible alteration in cortico-thalamic function [46] reduces slow wave synchronized neural activity in the cortex and the thalamus [47]. Cortical cooling in both sleeping and quiet awake animals eliminates silent states and allows neurons of the thalamic matrix to fire persistently even outside the period coinciding with the positive deflection of the surface EEG [47]. This firing behavior was present in approximately 50% and 40% of the units that fired in coincidence with the EEG in MCS and VS patients respectively, further suggesting the presence of damage to the cortico-thalamic functional axis. Our results are in agreement with studies showing that the degree of atrophy in the antero-dorsal limbic nuclei and in the medio-dorsal association nuclei of the thalamus is an important predictor of behavioral outcome after brain injury [48]. Our findings also support the results of fMRI investigations showing that thalamo-cortical network properties differ between healthy subjects and patients affected by DOC [49]. A significant reduction in the influence of the thalamus on the cortex, especially of the frontal lobe, seems correlated with the absence of conscious response [6,50]. Moreover, a diminished excitatory coupling between the motor thalamus and the primary motor cortex may be related to the lack of external responsiveness in covertly aware patients [51]. Thus both non-invasive studies of large scale cortico-thalamo-cortical connectivity performed by fMRI [6,49] that allow for control groups and larger populations but have intrinsic limitations in spatial and temporal resolution and our invasive study, based on single unit recording that has higher temporal and spatial resolution, but allows only a focused exploration of the thalamo-cortical network in a small number of subjects, suggest that disruption of thalamo-cortical connectivity is an important aspect of DOC.

Third, the differences in the number of active units, and in the distribution of the cells discharging synchronously with the EEG, together with the limitation of TC CFC to MCS provide further evidence that VS/UWS and MCS are not simply clinical definitions but truly represent two different, albeit pathological, brain states. In agreement with our findings the stronger evidence for a possible distinction between VS/UWS and MCS that is independent from behavioral data, comes from the study of the degree of persistence of the connections of the thalamus to the frontal and parietal lobes in the two conditions [52].

In accordance with the inclusion criteria of the CATS study all patients recorded had brain MRI showing bilateral absence of damage to the central and medial thalami and normal somatosensory and cortical auditory evoked potentials on at least one side [19]. These requirements were not matched by the majority (87.5%) of DOC patients initially evaluated for the study [19]. This was not unexpected since thalamic damage tends to be more extensive after traumatic injury compared to a non-traumatic one [8] and the majority of patients entering our study were post-traumatic [19]. So our results should apply to the part of the spectrum of VS/UWS and MCS patients of traumatic origin who have less damage and are, therefore, presumably, more likely to benefit from therapies.

Our works has also limitation since we lack a control group, it involves only three patients and the patients inhaled sevoflurane during the recordings.

Obvious ethical constrains and practical considerations common to most studies involving surgery or other invasive procedures make a control group (conscious patients) unfeasible. However, as discussed above our result significantly differ from the results of published microelectrode recordings in human and animal that were not affected by DOC and also differ according to the severity of DOC.

Our three patients, should be considered in the light of the numerosity of previous studies of thalamic stimulation in subject with DOC. Those studies were either based on one [21] or, when they involved a higher number of patients, the accrual periods were longer than 10 years [53]. A limited number of patients is also not uncommon in studies of patients affected by DOC even when non-invasive techniques are employed [51,54]. The light anesthesia that we introduced, according to an explicit request by the ethic review board of our institutions, is common in modern studies on DOC patients to reduce head movements, even when non-invasive MRI based studies are performed [10]. Moreover, we suspended all anaesthetic and sedative agents except for sevoflurane that was employed at 0.5–0.7% before single unit recordings. That is a very low concentration compared to 2% the MAC_90_ for this anesthetic vapor in humans [55]. In humans decreased thalamocortical connectivity as shown by simultaneous functional magnetic resonance and electroencephalography, has been described with sevoflurane only for concentrations of 2% and above [55]. Finally the effect of sevoflurane on single unit activity in the thalamus is known [38] and can not explain our findings.

Our results unveil for the first time the neurophysiological activity of the thalamus of VS/UWS and MCS at single-unit resolution pointing to unappreciated distinctions between these two states and states of reversible loss of consciousness occurring physiologically. Our data highlight important criteria that can be used in the neurophysiological classification of DOC and that could inform the development of new therapeutic approaches. Finally, our results could be helpful for future studies aimed at picking out subjects whose DOC is irreversible from others in whom it is not.

## Supporting information

S1 Fig Burst detection procedure. Burst were detected when the nonlinear filter (red line) passed a positive threshold (blue line). The occurrence of each spike is marked with green filled circles except for spikes occurring in bursts where circles are purple filled.
(TIF)

S1 Table Complete listing of all spiking and bursting neurons. Complete listing of all spiking and bursting thalamic neuron detected with the indications of their location in the hemisphere and the microelectrode tract. Neurons that fired in phase with the EEG phases are also indicated.
(XLSX)
