# Supplementary figures and images for: Single unit activities recorded in the thalamus and the overlying parietal cortex of subjects affected by disorders of consciousness
Source: PLoS One. 2018 Nov 7;13(11):e0205967. doi: 10.1371/journal.pone.0205967 (PMC6221278; doi:10.1371/journal.pone.0205967)

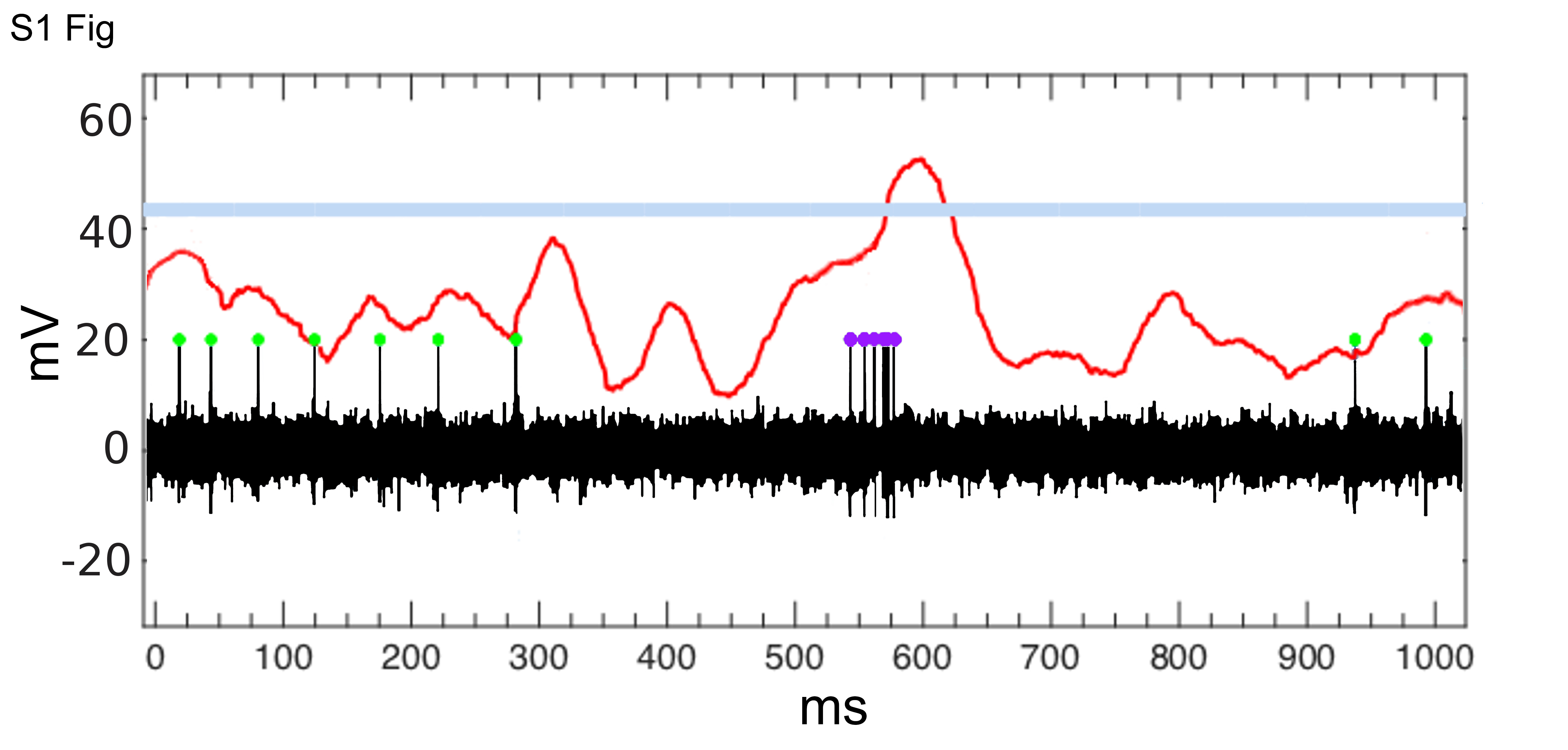

Supplement: S1 Fig — Burst were detected when the nonlinear filter (red line) passed a positive threshold (blue line). The occurrence of each spike is marked with green filled circles except for spikes occurring in bursts where circles are purple filled. (TIF) [file pone.0205967.s001.tif]
